# Metabolic effect of berberine–silymarin association: A meta‐analysis of randomized, double‐blind, placebo‐controlled clinical trials

**DOI:** 10.1002/ptr.6282

**Published:** 2019-01-10

**Authors:** Federica Fogacci, Davide Grassi, Manfredi Rizzo, Arrigo F.G. Cicero

**Affiliations:** ^1^ Medical and Surgical Sciences Department Alma Mater Studiorum University of Bologna Bologna Italy; ^2^ Department of Life, Health, and Environmental Sciences University of L'Aquila L'Aquila Italy; ^3^ Department of Internal Medicine and Medical Specialties University of Palermo Palermo Italy; ^4^ Italian Nutraceutical Society (SINut) Bologna Italy

**Keywords:** berberine, cholesterol, fasting plasma glucose, meta‐analysis, nutraceutical, silymarin

## Abstract

The aim of this study is to assess the impact of a combination of berberine and silymarin on serum lipids and fasting plasma glucose (FPG) through a systematic review of literature and meta‐analysis of the available randomized, double‐blind, placebo‐controlled clinical trials (RCTs). A systematic literature search in SCOPUS, PubMed‐Medline, ISI Web of Science, and Google Scholar databases was conducted up to October 2, 2018, in order to identify RCTs assessing changes in plasma concentrations of total cholesterol (TC), triglycerides (TG), high‐density lipoprotein cholesterol (HDL‐C), low‐density lipoprotein cholesterol (LDL‐C) and FPG during treatment with berberine and silymarin in combination. Two review authors independently extracted data on study characteristics, methods, and outcomes. Quantitative data synthesis was performed using a random‐effects model. We identified five eligible RCTs, with 497 subjects overall included. Berberine and silymarin combination treatment exerted a positive effect on TC (mean difference [MD]: −25.3, 95% CI [−39.2, −11.4] mg/dl; *p* < 0.001), TG (MD: −28, 95% CI [−35.3, −20.6] mg/dl; *p* < 0.001), HDL‐C [MD: 6, 95% CI [3.2, 8.8] mg/dl; *p* < 0.001), LDL‐C (MD: −29.1, 95% CI [−39.7, −18.6] mg/dl; *p* < 0.001), and FPG (MD: −7.5, 95% CI [−13, −1.9] mg/dl; *p* = 0.008). The present findings suggest that the coadministration of berberine and silymarin is associated with an advantageous improvement in lipid and glucose profile, suggesting the possible use of this nutraceutical combination in order to promote the cardiometabolic health.

## 1 BACKGROUND

Berberine (BBR) is a quaternary benzylisoquinoline alkaloid present in the root, rhizome, stem, fruit, and bark of different species of plants as *Coptis* (*Callosobruchus chinensis*, *japonica*), *Hydrastis* (Helicobacter canadensis), and *Berberis* (Berberis aristata, *vulgaris*, *croatica*; Liu, Zheng, Zhang, & Long, 2016). The lipid‐lowering effect of BBR is a relatively recent finding. It regulates plasma cholesterol levels essentially with two mechanisms. First, BBR inhibits the pro‐protein convertase subtilisin/kexin type 9 (PCSK9) through the ubiquitination and degradation of hepatocyte nuclear factor 1α, causing increased levels and a limited degradation of the hepatic LDL‐receptor. Second, BBR acts directly on the expression of LDL‐receptor by causing an up‐regulation of the receptors through a posttranscriptional mechanism that stabilizes their mRNA (activation of extracellular signal regulated kinases and jun amino‐terminal kinases dependent pathways; Abidi, Zhou, Jiang, & Liu, 2005; Li et al., 2009).

In addition, BBR has also some secondary mechanisms of action. As a matter of fact, recent studies have emphasized that it may be able to reduce the intestinal absorption of cholesterol, increasing its faecal excretion and promoting the hepatic cholesterol turnover and the formation of bile acids (Li et al., 2015). Moreover, BBR has been described to increase fatty acids oxidation and reduce the expression of lipogenic genes by activating the 5′ adenosine monophosphate‐activated protein kinase (Kim et al., 2009; Qiang et al., 2016). BBR also exerts a large number of additional functions by modulating glucose metabolism: Indeed, this alkaloid may increase insulin secretion, stimulate glycolysis, suppress adipogenesis, inhibit mitochondrial function, activate the adenosine monophosphate‐activated protein kinase pathway, and increase glycokinase activity. Furthermore, BBR has been reported to enhance the expression of glucose transporter‐4 and glucagon‐like peptide‐1 (Cicero & Tartagni, 2012). However, its oral bioavailability is lower than 1%, essentially for the poor intestinal absorption (around 56%), which is caused by a self‐particulate aggregation reducing the solubility in the gastrointestinal tract, by the low permeability of the molecule (Biopharmaceutical Classification System class III) and the intestinal and liver first‐pass metabolism (43.5% and 0.14%, respectively; Cicero et al., 2017). The effect of the intestinal first pass is still unclear, but it probably includes the enzymatic systems CYP2D6 and CYP3A4 in liver metabolism. Finally, BBR is also the substrate of the efflux pump P‐glycoprotein (P‐gp). Therefore, in recent years alternative approaches to increase the bioavailability of BBR have been studied, using permeability enhancers (sodium caprate, sodium deoxycholate, and chitosan), P‐gp inhibitors (silymarin), or modified release dosage forms (nanoemulsions, micelles, liposomes, and nanoparticles), with quite satisfactory results definitely (Mirhadi, Rezaee, & Malaekeh‐Nikouei, 2018).

The BBR–silymarin association use, in particular, is supported by a correct pharmacological background (Di Pierro et al., 2012; Di Pierro et al., 2013) and has been specifically tested in some well‐designed clinical trials (Derosa et al., 2013; Derosa, D'Angelo, & Maffioli, 2016; Derosa, Romano, D'Angelo, & Maffioli, 2015; Guarino et al., 2015; Guarino et al., 2017).

The aim of our meta‐analysis was to globally evaluate the lipid‐ and glucose‐lowering efficacy of the BBR–silymarin association, on the basis of the available randomized, double‐blind, placebo‐controlled clinical trials (RCTs).

## 2 METHODS

### 2.1 Search Strategy

The study was designed according to guidelines of the 2009 preferred reporting items for systematic reviews and meta‐analysis statement (Moher et al., 2009). PubMed‐Medline, Researchgate, SCOPUS, Google Scholar, and ISI Web of Science databases were searched, with no language restriction, using the following search terms: (“Berberine” OR “Berberol” OR “BBR” OR “Berberina”) AND (“Silymarin” OR “Silymarina” OR “Silimarina”) AND (“Clinical trial” OR “Clinical study” OR “Randomized” OR “Double‐blind”) AND (“Cholesterol” OR “Total cholesterol” OR “Total‐cholesterol” OR “TC” OR “T‐C” OR “Triglycerides” OR “TG” OR “Low‐density lipoprotein cholesterol” OR “LDL‐Cholesterol” OR “LDL‐C” OR “Fasting plasma glucose” OR “Plasma glucose” OR “Glycaemia”). The search was limited to studies in humans. The wild‐card term “*” was used to increase the sensitivity of the search strategy. Literature was searched from inception to October 2, 2018. The reference list of identified papers was manually checked for additional relevant articles.

### 2.2 Study selection criteria

Original studies were included in the meta‐analysis if they met the following inclusion criteria: (a) being a randomized trial with either parallel or cross‐over design and (b) investigating the impact of chronic BBR and silymarin supplementation on total cholesterol (TC), triglycerides (TG), low‐density lipoprotein cholesterol (LDL‐C), or fasting plasma glucose (FPG). Exclusion criteria were (a) lack of a control group for the combination of BBR and silymarin and (b) lack of sufficient information on baseline or follow‐up for at least one of the investigated parameters. Two authors independently reviewed all articles. Then, a third author arbitrated any discrepancies in including the studies in the meta‐analysis.

### 2.3 Data extraction

Data abstracted from the eligible studies were (a) first author's name; (b) year of publication; (c) study design; (d) treatment duration; (e) number of participants in the active and control group; (f) age, sex, and body mass index of study participants; and (g) baseline TC, TG, LDL‐C, and FPG.

### 2.4 Quality assessment

A systematic assessment of bias in the included studies was performed using the Cochrane criteria (Higgins & Green, 2010). The items utilized for the assessment of each study were as follows: adequacy of sequence generation, allocation concealment, blinding addressing of dropouts (incomplete outcome data), selective outcome reporting, and other probable sources of bias (Sahebkar et al., 2017).

### 2.5 Data synthesis

Meta‐analysis was entirely conducted using Comprehensive Meta‐Analysis V3 software (Biostat, NJ; Borenstein, Hedges, Higgins, & Rothstein, 2005). Net changes in the investigated parameters (change scores) were calculated by subtracting the value at baseline from the one after intervention, in the active‐treated group, and in the control one. *SD*s of the mean difference (MD) were obtained as reported by Follmann, Elliott, Suh, and Cutler (1992): *SD* = square root ([*SD*_pre‐treatment_]^2^ + [*SD*_post‐treatment_]^2^ – [2R × *SD*_pre‐treatment_ × *SD*_post‐treatment_]), assuming a correlation coefficient (*R*) = 0.5.

Studies' findings were combined using a random‐effect model due to the moderately high (>50%) heterogeneity, which was quantitatively assessed using the Higgins index (*I* ^2^; Melsen, Bootsma, Rovers, & Bonten, 2014). Finally, sensitivity analyses were conducted to account for risk of bias and a leave‐one‐out method was used (i.e., one study was removed at a time and the analysis repeated; Fogacci et al., 2018).

Effect sizes were expressed as MD and 95% confidence interval (CI); *p* ≤ 0.05 were considered as statistically significant for all tests.

### 2.6 Publication bias

Potential publication biases were explored using visual inspection of Begg's funnel plot asymmetry (Duval & Tweedie, 2000), Begg's rank correlation test, and Egger's regression test. In case of a significant result (*p* ≤ 0.05), the number of potentially missing studies required to make the *p* value nonsignificant was estimated by using the classical fail‐safe N method as another marker of publication bias.

## 3 RESULTS

### 3.1 Flow and characteristics of the included study

**Figure 1:**
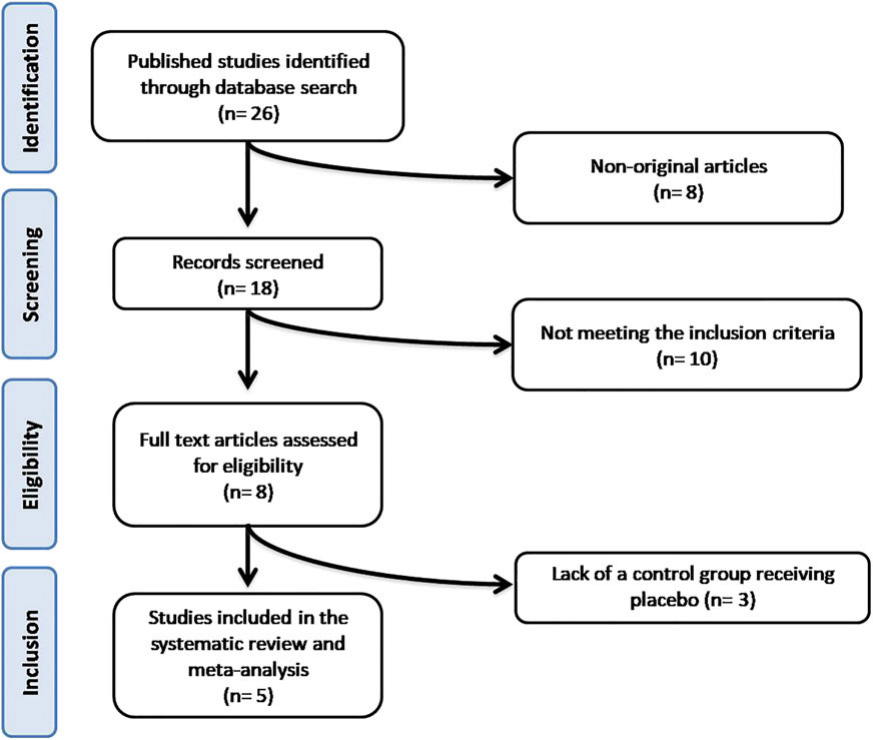
Flow chart of the number of studies identified and included into the meta‐analysis [Colour figure can be viewed at wileyonlinelibrary.com]

In summary, after several database searches, 26 published studies were identified and the abstracts reviewed. Of these, eight were nonoriginal article and were excluded. Then, other 10 studies were eliminated because they did not meet the inclusion criteria. Thus, eight full text articles were carefully assessed and reviewed. After assessment, three studies were excluded because lacking of a control group receiving placebo (*n* = 3; Appendix A). Finally, five studies were eligible and then included in the systematic review and meta‐analysis (Derosa et al., 2013; Derosa et al., 2015; Derosa et al., 2016; Guarino et al., 2015; Guarino et al., 2017). The study selection process is shown in Figure 1.

Data were pooled from five RCTs comprising 10 treatment arms, which included 497 subjects, with 251 subjects in the active treated arm and 246 subjects in the placebo one. All the included studies were published between 2013 and 2017. Selected trials were all designed per parallel groups. Baseline anthropometric, clinical, and biochemical characteristics of the evaluated studies are presented in Table 1.

**Table 1:** Baseline characteristics of the studies included in the meta‐analysis. Data are reported as mean ± standard deviation, unless otherwise specified

First author (year)	Study design	Treatment duration	Participants (*n*)	Study group	Age (years)	Male, *n* (%)	BMI (kg/m^2^)	TC (mg/dl)	LDL‐C (mg/dl)	TG (mg/dl)	FPG (mg/dl)
Guarino et al., 2017	Randomized, double‐blind, placebo‐controlled, parallel group clinical study	52 weeks	68	BBR 1,000 mg/day, silymarin 210 mg/day	56 ± 8	28 (41)	34 ± 4	230 ± 18	107 ± 16	198 ± 18	131 ± 22
			68	Placebo	55 ± 9	28 (41)	34 ± 5	237 ± 15	109 ± 14	201 ± 15	139 ± 18
Derosa et al., 2016	Randomized, double‐blind, placebo‐controlled, parallel group clinical study	6 months	41	BBR 500 mg/day, silymarin 105 mg/day	30.7 ± 8.1	19 (46)	22.9 ± 1.9	209.1 ± 22.4	136.9 ± 19.2	124.3 ± 35.8	148.3 ± 31.7
			44	Placebo	29.8 ± 7.2	20 (45)	22.6 ± 1.8	204.8 ± 17.8	133.7 ± 18.4	119.5 ± 31.6	141.8 ± 28.4
Derosa et al., 2015	Randomized, double‐blind, placebo‐controlled, parallel group clinical study	6 months	66	BBR 500 mg/day, silymarin 105 mg/day	57.8 ± 12.6	32 (48)	28.8 ± 1.1	188.6 ± 30.9	129.2 ± 11.5	92.8 ± 36.7	92.8 ± 6.1
			62	Placebo	57.9 ± 12.9	31 (50)	29.5 ± 1.3	184.5 ± 28.3	124.6 ± 10.6	95.3 ± 38.2	91.7 ± 5.9
Guarino et al., 2015	Randomized, double‐blind, placebo‐controlled, parallel groups clinical study	6 months	25	BBR 500 mg/day, silymarin 105 mg/day	54 ± 5	14 (56)	34 ± 3	230 ± 14	NA	NA	137 ± 22
			25	Placebo	56 ± 7	13 (52)	34 ± 2	235 ± 13	NA	NA	141 ± 19
Derosa et al., 2013	Randomized, double‐blind, placebo‐controlled, parallel group clinical study	3 months	51	BBR 1,000 mg/day, silymarin 210 mg/day	52 ± 10.5	27 (53)	26.2 ± 1.7	212 ± 11.2	151 ± 9.3	99.6 ± 26.5	84.1 ± 8.2
			47	Placebo		24 (51)	27 ± 1.5	212.4 ± 11.5	151.5 ± 9.3	97.8 ± 24.9	84.4 ± 8.4

*Note*. BBR: berberine; BMI: body mass index; FPG: fasting plasma glucose; *n*: subjects; NA: not available; TC: total cholesterol; TG: triglycerides; LDL‐C: low‐density lipoprotein cholesterol.

### 3.2 Risk of bias assessment

All the included studies were characterized by sufficient information regarding sequence generation, allocation concealment, and personnel and outcome assessments and showed low risk of bias because of incomplete outcome data and selective outcome reporting. Details of the quality of bias assessment are reported in Table 2.

**Table 2:** Quality of bias assessment of the included studies according to Cochrane guidelines

Author	Sequence generation	Allocation concealment	Blinding of participants, personnel, and outcome assessment	Incomplete outcome data	Selective outcome reporting	Other potential threats to validity
Guarino et al., 2017	L	L	U	L	L	U
Derosa et al., 2016	L	L	L	L	L	L
Derosa et al., 2015	L	L	L	L	L	L
Guarino et al., 2015	L	L	U	L	H	U
Derosa et al., 2013	L	L	L	L	L	L

*Note*. L: low risk of bias; H: high risk of bias; U: unclear risk of bias.

### 3.3 Effect of BBR and silymarin on plasma lipids and glucose concentrations

**Figure 2:**
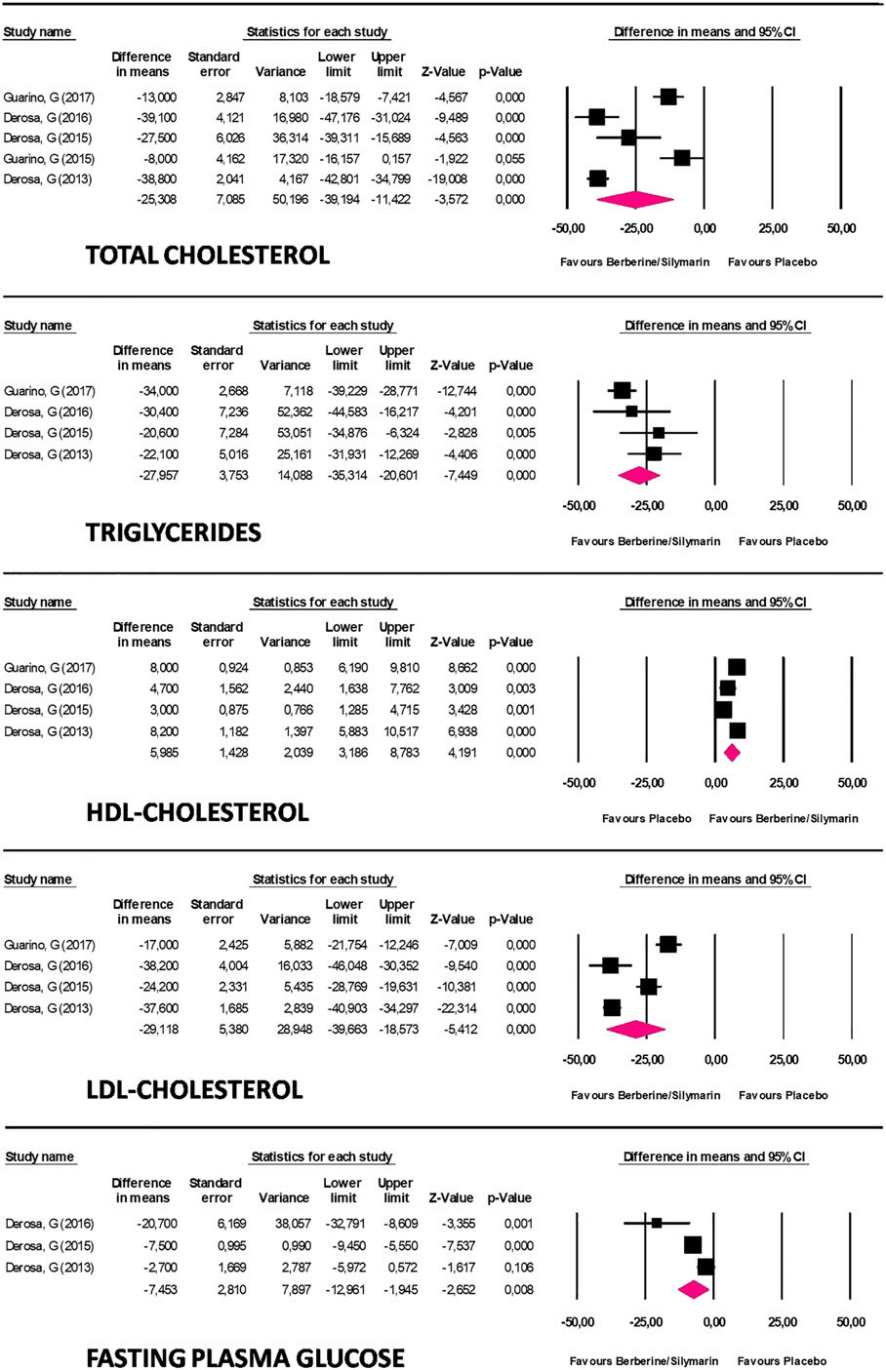
Forest plot detailing mean differences and 95% confidence intervals for the studies included in the meta‐analysis [Colour figure can be viewed at wileyonlinelibrary.com]

**Figure 3:**
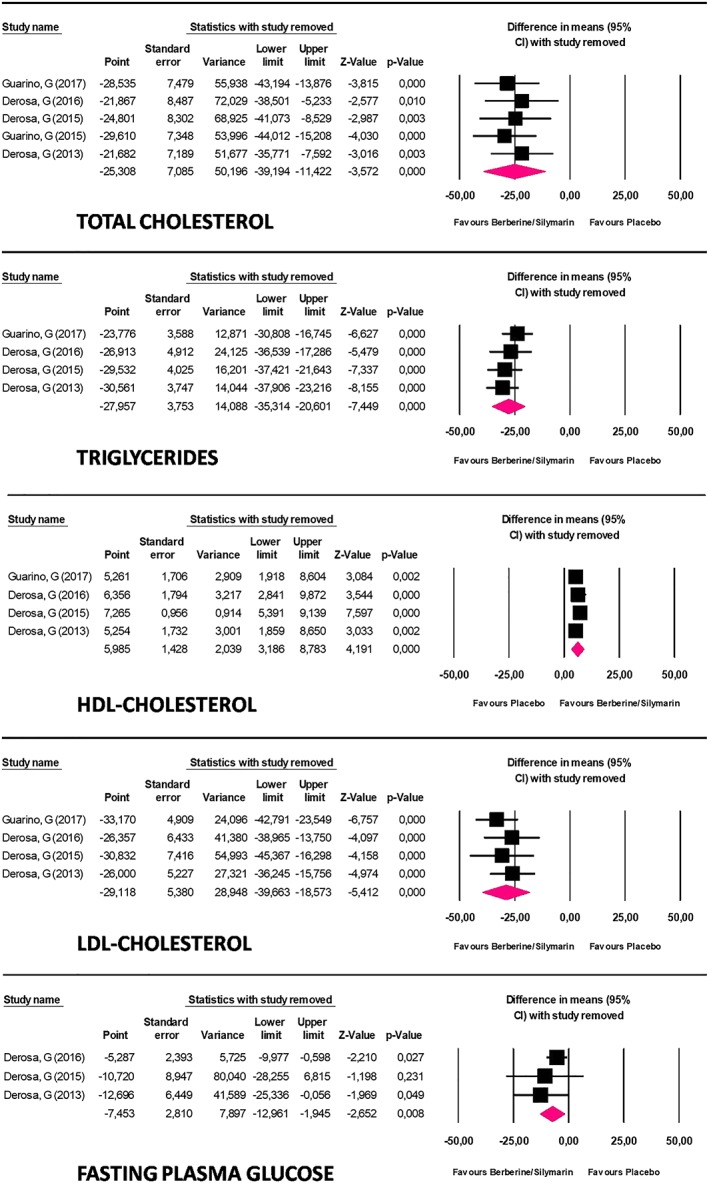
Plot showing leave‐one‐out sensitivity analysis [Colour figure can be viewed at wileyonlinelibrary.com]

The effect of BBR and silymarin on plasma concentrations of TC, TG, high‐density lipoprotein cholesterol (HDL‐C), LDL‐C, and FPG was reported in five, four, four, four, and three studies, respectively. The combined supplementation was found to significantly reduce TC (MD: −25.3, 95% CI [−39.2, −11.4] mg/dl; *p* < 0.001; *I* ^2^ = 95%), TG (MD: −28, 95% CI [−35.3, −20.6] mg/dl; *p* < 0.001; *I* ^2^ = 53%), HDL‐C (MD: 6, 95% CI [3.2, 8.8] mg/dl; *p* < 0.001; *I* ^2^ = 85%), LDL‐C (MD: −29.1, 95% CI [−39.7, −18.6] mg/dl; *p* < 0.001; *I* ^2^ = 95%), and FPG (MD: −7.5, 95% CI [−13, −1.9] mg/dl; *p* = 0.008; *I* ^2^ = 83%; Figure 2). These results were robust in the leave‐one‐out sensitivity analysis (Figure 3).

### 3.4 Publication biases

**Figure 4:**
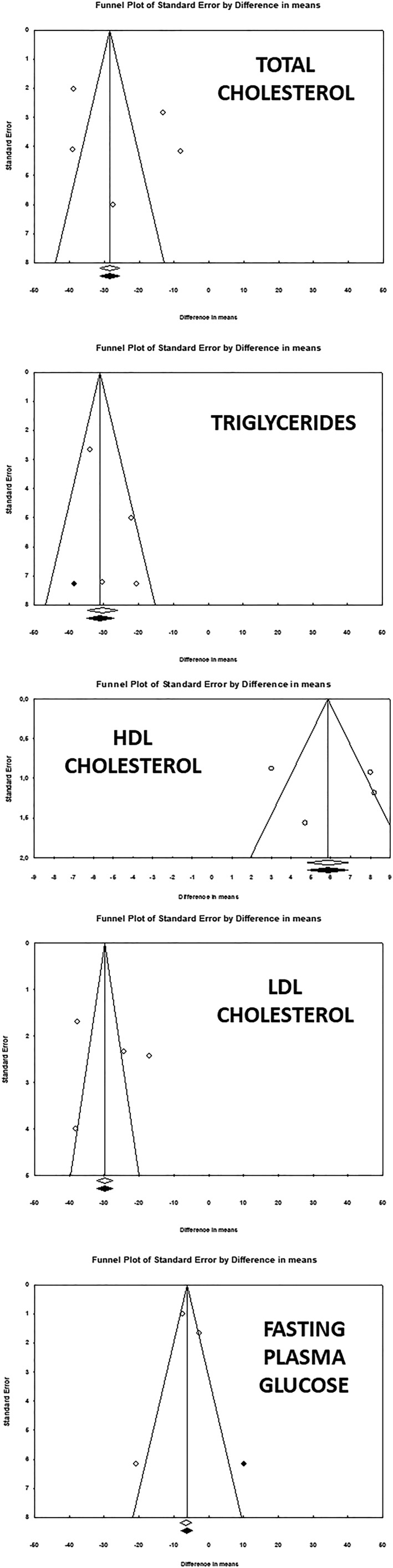
Funnel plot detailing publication biases in the studies included in the meta‐analysis

The funnel plots of standard error by effect size (MD) were symmetric, suggesting no publication biases in the meta‐analysis (Figure 4). The absence of publication biases was confirmed by the Egger's regression and the Begg's rank correlation. The fail‐safe N test showed that 403 studies would be needed to bring on TC the effect size to a nonsignificant level (*p* > 0.05), 149 studies would be needed to bring on TG the effect size to a nonsignificant level, 123 studies would be needed to bring on HDL‐C the effect size to a nonsignificant level, 628 studies would be needed to bring on LDL‐C the effect size to a nonsignificant level, and 38 studies would be needed to bring on FPG the effect size to a nonsignificant level.

## 4 DISCUSSION

At the best of our knowledge, the current systematic review and meta‐analysis is the first one to comprehensively analyse evidences from RCTs on the metabolic effect of berberine–silymarin association.

Recently, an exponentially growing body of evidence has supported the hypothesis than the use of a combined nutraceutical compound can exert a greater preventive and therapeutic success than a single biomolecule, because of both additive and synergistic effects of each individual constituent (Cicero et al., 2017; Cicero, Colletti, Bajraktari, et al., 2017; Cicero, Fogacci, & Colletti, 2017).

BBR lipid‐lowering efficacy in humans is well‐known and definitely confirmed by a meta‐analysis of 27 clinical studies with overall 2,569 participants (Lan et al., 2015). In comparison with that meta‐analysis, our findings show that the addition of silymarin to BBR is able to improve its positive effect on lipid and glucose metabolism in humans, allowing for the administration of lower doses of BBR and accordingly reducing the associated risk of gastrointestinal discomfort which is demonstrably dose related (Caliceti, Rizzo, & Cicero, 2015; Cicero & Baggioni, 2016). As a matter of fact, considerations on tolerability of low doses of BBR may also have important clinical implications, because it is well known that hypercholesterolemia is an asymptomatic clinical condition in which adherence and persistence on prescribed lipid‐lowering medications are relatively low (Malo et al., 2017), and discontinuation rates are even higher in presence of adverse events or drug reactions (Banach et al., 2018).

Furthermore, it could be argued that silymarin per se could exert some additive effects on lipid and glucose parameters. As a matter of fact, silymarin, a complex of flavonolignans from the fruit *Sylibum marianum*, has shown in preclinical test to inhibit cholesterol acyltransferase and HMG‐CoA reductase activity and improve LDL‐C uptake by the liver, definitely reducing cholesterol absorption and lipoprotein biosynthesis (Skottová & Krecman, 1998; Sobolová, Skottová, Vecera, & Urbánek, 2006). Recently, additional antioxidant properties have been described for silymarin, highlighting the advantages of beneficial silymarin supplementation on hepatic function and, of consequence, on glucose and lipid metabolism (Surai, 2015). In humans, silymarin has been described to ameliorate glycemic control, with a reduction in both fasting insulin and exogenous insulin requirements in insulin‐treated patients with type 2 diabetes and hepatic cirrhosis (Voroneanu, Nistor, Dumea, Apetrii, & Covic, 2016). However, the polyphenolic substances constituting silymarin (silybin, isosilybin, silydianin, and silychristin) have poor water solubility and very low bioavailability in humans (Calani, Brighenti, Bruni, & Del Rio, 2012). Therefore, it is more likely that silymarin improves BBR oral bioavailability by directly interacting with P‐gp (Gazák, Walterová, & Kren, 2007), rather than affect itself glucose and lipid metabolism in humans.

Certainly, the present meta‐analysis has some limitations. First, among the eligible RCTs was found a moderate to high degree of heterogeneity, which may be due to differences in the intervention duration, sample size, and daily dose of the treatment. Second, almost all the included trials have short duration, so that further studies are needed to determine whether these short‐term *effects are maintained with long‐term*. Finally, the included studies enrolled only adult subjects, so that we cannot directly infer our results to children and elderly. However, our findings suggest a potential way to improve at the same time the lipid and glucose profile. This is of great importance especially considering the high prevalence of diabetes among hypercholesterolemic subjects and the increased risk of atherosclerotic‐related diseases in diabetic patients with hypercholesterolemia (Besseling, Kastelein, Defesche, Hutten, & Hovingh, 2015; Katakami, 2018).

In conclusion, the favourable effect of BBR–silymarin association emerging from the current meta‐analysis suggests its possible use in order to promote cardiovascular health.

## FUNDING INFORMATION

This paper was written independently.

## CONFLICT OF INTEREST

Authors have no conflict of interest to declare.

## APPENDIX A

### STUDIES NOT MEETING THE INCLUSION CRITERIA (EXCLUDED FROM THE META‐ANALYSIS)

- Lack of a control group receiving placebo (*n* = 3)

1. Di Pierro F., Bellone I., Rapacioli G., & Putignano P. (2015). Clinical role of a fixed combination of standardized *Berberis aristata* and Silybum marianum extracts in diabetic and hypercholesterolemic patients intolerant to statins. *Diabetes, Metabolic Syndrome and Obesity,* 8, 89–96. doi: 10.2147/DMSO.S78877.
2. Di Pierro F., Putignano P., Villanova N., Montesi L., Moscatiello S., & Marchesini G. (2013). Preliminary study about the possible glycemic clinical advantage in using a fixed combination of *Berberis aristata* and Silybum marianum standardized extracts versus only Berberis aristata in patients with type 2 diabetes. *Clinical Pharmacology: Advances and Applications,* 5, 167–174. doi: 10.2147/CPAA.S54308.
3. Di Pierro F., Villanova N., Agostini F., Marzocchi R., Soverini V., & Marchesini G. (2012). Pilot study on the additive effects of berberine and oral type 2 diabetes agents for patients with suboptimal glycemic control. *Diabetes, Metabolic Syndrome and Obesity,* 5, 213–217. doi: 10.2147/DMSO.S33718.

## References

[ptr6282-bib-0001] Abidi, P. , Zhou, Y. , Jiang, J. D. , & Liu, J. (2005). Extracellular signal‐regulated kinase‐dependent stabilization of hepatic low‐density lipoprotein receptor mRNA by herbal medicine berberine. Arteriosclerosis, Thrombosis, and Vascular Biology, 25(10), 2170–2176. 10.1161/01.ATV.0000181761.16341.2b 16100034

[ptr6282-bib-0002] Banach, M. , Patti, A. M. , Giglio, R. V. , Cicero, A. F. G. , Atanasov, A. G. , Bajraktari, G. , … International Lipid Expert Panel (ILEP) (2018). The role of nutraceuticals in statin intolerant patients. Journal of the American College of Cardiology, 72(1), 96–118. 10.1016/j.jacc.2018.04.040 29957236

[ptr6282-bib-0003] Besseling, J. , Kastelein, J. J. , Defesche, J. C. , Hutten, B. A. , & Hovingh, G. K. (2015). Association between familial hypercholesterolemia and prevalence of type 2 diabetes mellitus. JAMA, 313(10), 1029–1036. 10.1001/jama.2015.1206 25756439

[ptr6282-bib-0004] Borenstein, M. , Hedges, L. , Higgins, J. , & Rothstein, H. (2005). Comprehensive meta‐analysis (3^rd^ version. Englewood, NJ: Biostat. 104

[ptr6282-bib-0005] Calani, L. , Brighenti, F. , Bruni, R. , & Del Rio, D. (2012). Absorption and metabolism of milk thistle flavanolignans in humans. Phytomedicine, 20(1), 40–46. 10.1016/j.phymed.2012.09.004 23072776

[ptr6282-bib-0006] Caliceti C. , Rizzo P. , & Cicero A.F. (2015). Potential benefits of berberine in the management of perimenopausal syndrome. Oxidative Medicine and Cellular Longevity. 2015, 723093 10.1155/2015/723093, 1, 9. 25785174 PMC4346702

[ptr6282-bib-0007] Cicero, A. F. , & Baggioni, A. (2016). Berberine and its role in chronic disease. Advances in Experimental Medicine and Biology, 928, 27–45. 10.1007/978-3-319-41334-1_2 27671811

[ptr6282-bib-0008] Cicero, A. F. , & Tartagni, E. (2012). Antidiabetic properties of berberine: From cellular pharmacology to clinical effects. Hospital Practice, 40(2), 56–63. 10.3810/hp.2012.04.970 22615079

[ptr6282-bib-0009] Cicero, A. F. G. , Colletti, A. , Bajraktari, G. , Descamps, O. , Djuric, D. M. , Ezhov, M. , … Banach, M. (2017). Lipid lowering nutraceuticals in clinical practice: Position paper from an International Lipid Expert Panel. Archives of Medical Science, 13(5), 965–1005. 10.5114/aoms.2017.69326 28883839 PMC5575230

[ptr6282-bib-0010] Cicero, A. F. G. , Fogacci, F. , & Colletti, A. (2017). Food and plant bioactives for reducing cardiometabolic disease risk: An evidence based approach. Food & Function, 8(6), 2076–2088. 10.1039/c7fo00178a 28541356

[ptr6282-bib-0011] Cicero, A. F. G. , Fogacci, F. , Rosticci, M. , Parini, A. , Giovannini, M. , Veronesi, M. , … Borghi, C. (2017). Effect of a short‐term dietary supplementation with phytosterols, red yeast rice or both on lipid pattern in moderately hypercholesterolemic subjects: A three‐arm, double‐blind, randomized clinical trial. Nutrition and Metabolism, 14, 61 10.1186/s12986-017-0214-2 29021813 PMC5613479

[ptr6282-bib-0012] Derosa, G. , Bonaventura, A. , Bianchi, L. , Romano, D. , D'Angelo, A. , Fogari, E. , & Maffioli, P. (2013). *Berberis aristata*/*Silybum marianum* fixed combination on lipid profile and insulin secretion in dyslipidemic patients. Expert Opinion on Biological Therapy, 13(11), 1495–1506. 10.1517/14712598.2013.832751 23971720

[ptr6282-bib-0013] Derosa, G. , D'Angelo, A. , & Maffioli, P. (2016). The role of a fixed *Berberis aristata*/*Silybum marianum* combination in the treatment of type 1 diabetes mellitus. Clinical Nutrition, 35(5), 1091–1095. 10.1016/j.clnu.2015.08.004 26384091

[ptr6282-bib-0014] Derosa, G. , Romano, D. , D'Angelo, A. , & Maffioli, P. (2015). *Berberis aristata* combined with *Silybum marianum* on lipid profile in patients not tolerating statins at high doses. Atherosclerosis, 239(1), 87–92. 10.1016/j.atherosclerosis.2014.12.043 25577665

[ptr6282-bib-0015] Di Pierro, F. , Putignano, P. , Villanova, N. , Montesi, L. , Moscatiello, S. , & Marchesini, G. (2013). Preliminary study about the possible glycemic clinical advantage in using a fixed combination of *Berberis aristata* and *Silybum marianum* standardized extracts versus only *Berberis aristata* in patients with type 2 diabetes. Clinical Pharmacology: Advances and Applications, 5, 167–174. 10.2147/CPAA.S54308 24277991 PMC3838471

[ptr6282-bib-0016] Di Pierro, F. , Villanova, N. , Agostini, F. , Marzocchi, R. , Soverini, V. , & Marchesini, G. (2012). Pilot study on the additive effects of berberine and oral type 2 diabetes agents for patients with suboptimal glycemic control. Diabetes, Metabolic Syndrome and Obesity, 5, 213–217. 10.2147/DMSO.S33718. PMC3422905 22924000

[ptr6282-bib-0017] Duval, S. , & Tweedie, R. (2000). Trim and fill: a simple funnel plot–based method of testing and adjusting for publication bias in meta‐analysis. Biometrics, 56, 455–463. 10.1111/j.0006-341X.2000.00455.x 10877304

[ptr6282-bib-0018] Fogacci, F. , Tocci, G. , Presta, V. , Fratter, A. , Borghi, C. , & Cicero, A. F. G. (2018). Effect of resveratrol on blood pressure: A systematic review and meta‐analysis of randomized, controlled, clinical trials. Critical Reviews in Food Science and Nutrition, 10.1080/10408398.2017.1422480. [Epub ahead of print], 1–14. 29359958

[ptr6282-bib-0019] Follmann, D. , Elliott, P. , Suh, I. , & Cutler, J. (1992). Variance imputation for overviews of clinical trials with continuous response. Journal of Clinical Epidemiology, 45, 769–773. 10.1016/0895-4356(92)90054-Q 1619456

[ptr6282-bib-0020] Gazák, R. , Walterová, D. , & Kren, V. (2007). Silybin and silymarin—New and emerging applications in medicine. Current Medicinal Chemistry, 14(3), 315–338. 10.2174/092986707779941159 17305535

[ptr6282-bib-0021] Guarino, G. , Della, C. T. , Sofia, M. , Carbone, L. , Marino, G. , Martedi, E. , & Gentile, S. (2015). Effetti metabolici dell'associazione berberina‐silmarina vs placebo in diabetici di tipo 2 obesi, ipercolesterolemici. Il Giornale di ADM, 18, 188–191. [Italian]

[ptr6282-bib-0022] Guarino, G. , Strollo, F. , Carbone, L. , Della, C. T. , Letizia, M. , Marino, G. , & Gentile, S. (2017). Bioimpedance analysis, metabolic effects and safety of the association Berberis aristata/Bilybum marianum: A 52‐week double‐blind, placebo‐controlled study in obese patients with type 2 diabetes. Journal of Biological Regulators and Homeostatic Agents, 31(2), 495–502. 28685558

[ptr6282-bib-0023] Higgins, J. , & Green, S. (2010). Cochrane handbook for systematic reviews of interventions. Version 5.0.2.2009. Chichester, UK: John Wiley and Sons Ltd. Ref Type: Report

[ptr6282-bib-0024] Katakami, N. (2018). Mechanism of development of atherosclerosis and cardiovascular disease in diabetes mellitus. Journal of Atherosclerosis and Thrombosis, 25(1), 27–39. 10.5551/jat.RV17014 28966336 PMC5770221

[ptr6282-bib-0025] Kim, W. S. , Lee, Y. S. , Cha, S. H. , Jeong, H. W. , Choe, S. S. , Lee, M. R. , … Kim, J. B. (2009). Berberine improves lipid dysregulation in obesity by controlling central and peripheral AMPK activity. American Journal of Physiology. Endocrinology and Metabolism, 296(4), E812–E819. 10.1152/ajpendo.90710.2008 19176354

[ptr6282-bib-0026] Lan, J. , Zhao, Y. , Dong, F. , Yan, Z. , Zheng, W. , Fan, J. , & Sun, G. (2015). Meta‐analysis of the effect and safety of berberine in the treatment of type 2 diabetes mellitus, hyperlipemia and hypertension. Journal of Ethnopharmacology, 161, 69–81. 10.1016/j.jep.2014.09.049 25498346

[ptr6282-bib-0027] Li, H. , Dong, B. , Park, S. W. , Lee, H. S. , Chen, W. , & Liu, J. (2009). Hepatocyte nuclear factor 1alpha plays a critical role in PCSK9 gene transcription and regulation by the natural hypocholesterolemic compound berberine. Journal of Biological Chemistry, 284(42), 28885–28895. 10.1074/jbc.M109.052407 19687008 PMC2781434

[ptr6282-bib-0028] Li, X. Y. , Zhao, Z. X. , Huang, M. , Feng, R. , He, C. Y. , Ma, C. , … Jiang, J. D. (2015). Effect of Berberine on promoting the excretion of cholesterol in high‐fat diet‐induced hyperlipidemic hamsters. Journal of Translational Medicine, 13, 278 10.1186/s12967-015-0629-3 26310319 PMC4549888

[ptr6282-bib-0029] Liu, C. S. , Zheng, Y. R. , Zhang, Y. F. , & Long, X. Y. (2016). Research progress on berberine with a special focus on its oral bioavailability. Fitoterapia, 109, 274–282. 10.1016/j.fitote.2016.02.001 26851175

[ptr6282-bib-0030] Malo, S. , Aguilar‐Palacio, I. , Feja, C. , Lallana, M. J. , Rabanaque, M. J. , Armesto, J. , & Menditto, E. (2017). Different approaches to the assessment of adherence and persistence with cardiovascular‐disease preventive medications. Current Medical Research and Opinion, 33(7), 1329–1336. 10.1080/03007995.2017.1321534 28422521

[ptr6282-bib-0031] Melsen, W. G. , Bootsma, M. C. , Rovers, M. M. , & Bonten, M. J. (2014). The effects of clinical and statistical heterogeneity on the predictive values of results from meta‐analyses. Clinical Microbiology and Infection, 20, 123–129. 10.1111/1469-0691.12494 24320992

[ptr6282-bib-0032] Mirhadi, E. , Rezaee, M. , & Malaekeh‐Nikouei, B. (2018). Nano strategies for berberine delivery, a natural alkaloid of Berberis. Biomedicine & Pharmacotherapy, 104, 465–473. 10.1016/j.biopha.2018.05.067 29793179

[ptr6282-bib-0033] Moher, D. , Liberati, A. , Tetzlaff, J. , Altman, D. G. , & PRISMA Group (2009). Preferred reporting items for systematic reviews and meta‐analyses: The PRISMA statement. British Medical Journal, 339, b2535 10.1136/bmj.b2535 21603045 PMC3090117

[ptr6282-bib-0034] Qiang, X. , Xu, L. , Zhang, M. , Zhang, P. , Wang, Y. , Wang, Y. , … Zhang, Y. (2016). Demethyleneberberine attenuates non‐alcoholic fatty liver disease with activation of AMPK and inhibition of oxidative stress. Biochemical and Biophysical Research Communications, 472(4), 603–609. 10.1016/j.bbrc.2016.03.019 26970305

[ptr6282-bib-0035] Sahebkar, A. , Pirro, M. , Banach, M. , Mikhailidis, D. P. , Atkin, S. L. , & Cicero, A. F. G. (2017). Lipid‐lowering activity of artichoke extracts: A systematic review and meta‐analysis. Critical Reviews in Food Science and Nutrition, 1–8. 10.1080/10408398.2017.1332572 28609140

[ptr6282-bib-0036] Skottová, N. , & Krecman, V. (1998). Silymarin as a potential hypocholesterolaemic drug. Journal of Physiological Research, 47(1), 1–7. 9708694

[ptr6282-bib-0037] Sobolová, L. , Skottová, N. , Vecera, R. , & Urbánek, K. (2006). Effect of silymarin and its polyphenolic fraction on cholesterol absorption in rats. Pharmacological Research, 53(2), 104–112. 10.1016/j.phrs.2005.09.004 16275123

[ptr6282-bib-0038] Surai, P. F. (2015). Silymarin as a natural antioxidant: An overview of the current evidence and perspectives. Antioxidants (Basel), 4(1), 204–247. 10.3390/antiox4010204 26785346 PMC4665566

[ptr6282-bib-0039] Voroneanu, L. , Nistor, I. , Dumea, R. , Apetrii, M. , & Covic, A. (2016). Silymarin in type 2 diabetes mellitus: A systematic review and meta‐analysis of randomized controlled trials. Journal Diabetes Research, 2016, 5147468. 10.1155/2016/5147468, –10. PMC4908257 27340676

